# Evaluation of commercial extension plates for the ACR CT accreditation phantom

**DOI:** 10.1120/jacmp.v17i1.5785

**Published:** 2016-01-08

**Authors:** Kerry A. Greene‐Donnelly, Kent M. Ogden

**Affiliations:** ^1^ Medical Imaging Sciences SUNY Upstate Medical University Syracuse New York USA; ^2^ Radiology, SUNY Upstate Medical University Syracuse New York USA

**Keywords:** computed tomography, accreditation, quality control

## Abstract

The American College of Radiology (ACR) Computed Tomography (CT) Accreditation Program requires submission of phantom scans acquired with the ACR accreditation phantom. There is a known issue with some wide‐beam scanners in which the Hounsfield unit (HU) value of water may be correct when using the scanner manufacturer's phantom, but will be out of range in some scan modes when scanning the accreditation phantom. The phantom manufacturer has developed a product known as Extension Plates to eliminate the water HU value issue. The purpose of this technical note is to evaluate the effectiveness of the Extension Plates in alleviating the water HU issue. The ACR phantom was scanned on nine different CT scanners representing four CT manufacturers at eight different facilities. Scanner models included 16‐ and 64‐channel geometries from each manufacturer. All scanners passed routine daily water HU testing per the manufacturer's instructions. The accreditation phantom was scanned in helical and axial modes both with and without the Extension Plates present. Regions of interest were placed on the linearity test objects as well as the water HU test object in Module 1 of the phantom. Mean values were recorded and compared with the acceptable ranges specified by the ACR accreditation phantom testing instructions. Water HU values failed for one scanner model when scanned in helical mode using the widest collimation available and the Extension Plates were not present. All other scanner models passed the water HU linearity test with or without the Extension Plates in both axial and helical scan modes. Three of the four manufacturers tested failed the linearity test for different materials. The presence of the Extension Plates only affected the HU measurement for the water test object.

PACS number(s): 87.57Q, 87.57C

## I. INTRODUCTION

The Gammex 464 phantom (Gammex Inc, Middleton, WI) for CT was developed to assess several quality indicators with the use of a single phantom. Its use is required as part of the American College of Radiology CT Accreditation Program (CTAP) http://www.acr.org/~/media/ACR/Documents/Accreditation/CT/PhantomTestingInstruction.pdf.^(1)^ The phantom evaluates Hounsfield unit (HU) accuracy/linearity, image thickness, image noise, positioning accuracy, low‐contrast resolution, high‐contrast resolution, geometric in‐plane measurements, and uniformity.^(2)^ We refer to the Model 464 phantom here as the ACR phantom. It is recognized by the ACR that the HU values of some of the test objects in Module 1 of the ACR phantom are not within acceptable ranges. As discussed in the ACR Accreditation FAQ, a water phantom or a CTDI phantom may be placed adjacent to the Gammex 464 phantom to improve the HU assessment.^(3)^ The effects of scatter radiation in conjunction with wide beam widths have been proposed as a possible cause of inaccurate HU assessment in the Gammex 464 phantom.^(4)^

We evaluated the utility of optional Extension Plates (Gammex 464‐EXTPLT‐KIT), designed to improve the accuracy of HU measurement in solid water. We investigated scanners from four different vendors: GE Medical Systems (Waukesha, WI), Philips Healthcare (Andover, MA), Siemens Healthcare (Erlangen, Germany), and Toshiba Medical Systems (Tochigi‐ken, Japan), representing two different scanner geometries (64‐ and 16‐ channel) at eight independent clinical sites (nine scanners total). The 64‐channel scanners investigated were the GE LightSpeed VCT, Philips Brilliance CT, Siemens Somatom AS, and Toshiba Aquilion 64. The 16‐channel scanners investigated were GE LightSpeed Ultra, Philips Brilliance, Siemens Somatom Sensation 16, and a Toshiba Aquilion 16.

## II. MATERIALS AND METHODS

**Figure 1:**
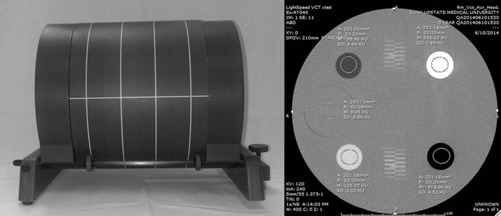
Gammex 464 phantom positioned on extended cradle with Extension Plates (left), CT image obtained in Module 1. Image acquired using a GE VCT 64 channel scanner with Extension Plates, showing a passing Solid Water HU (right).

For each scanner, a water HU assessment was performed per the vendor‐specific instructions to verify accurate scanner calibration prior to the ACR phantom scans. The ACR phantom was scanned using an axial head and helical abdominal protocol (1, 2). A scan was performed with and without the Extension Plates (Fig. 1) for each protocol on each scanner. In helical mode, the widest beam width available on each scanner was used.

Regions of interest (ROIs) were placed in the Module 1 linearity test objects and mean HU values were recorded (3, 4). All ROI measurements followed the ACR accreditation phantom testing guidelines.^(2)^ ROIs were placed on the centermost image of Module 1 and were approximately 200 mm^2^ in area. Each ROI was placed in the center of the material being measured for the most accurate assessment of HU (Fig.1. The materials measured included polyethylene, solid water, acrylic, bone, and air. Acceptable HU ranges for each material measured are listed in Table 5.

**Table 1:** Head protocols.

	*GE*		*Philips*		*Siemens*		*Toshiba*	
*Geometry*	*64*	*16*	*64*	*16*	*64*	*16*	*64*	*16*
kVp	120	120	120	120	120	120	120	120
mAs	350	350	350	350	350	350	350	350
FOV (mm)	210	210	210	210	210	210	210	210
Slice (mm)	5	5	5	3	5	5	4	4
Kernel	std	std	std UB	UB	H31s	H41s	FC25	FC69
Beam (mm)	40	20	10	12	40	10	16	16

**Table 2:** Abdomen protocols.

	*GE*		*Philips*		*Siemens*		*Toshiba*	
*Geometry*	*64*	*16*	*64*	*16*	*64*	*16*	*64*	*16*
kVp	120	120	120	120	120	120	120	120
mAs	240	240	240	223	240	240	240	240
FOV (mm)	210	210	210	210	210	210	210	210
Slice (mm)	5	5	5	5	5	5	5	5
Kernel	std	std	std B	C	B40f	B31s	FC13	FC18
Pitch	1.35	1.35	1.36	1.19	1.3	1	1.48	1.48
Beam (mm)	40	20	40	24	40	24	32	16

**Table 3:** Axial head protocol HU values with and without (W/O) Extension Plates.

	*Polyethylene*	*Solid Water*	*Acrylic*	*Bone*	*Air*
*64 Channel Scanners*					
GE(W/O)	−90.82	4.94	121.63	983.07^a^	−974.7
GE	−91.84	3.3	120.12	977.11^a^	−973.01
Philips(W/O)	−89.1	4.1	129.6	850.7	−999
Philips	−89.5	4.2	129	851.1	−998.8
Siemens(W/O)	−89.34	0.74	126.73	873.12	−994.97
Siemens	−90.09	0.8	126.45	872.41	−995.31
Toshiba(W/O)	−96	−1.5	113.7	919.2	−973.6
Toshiba	−91.9	3.9	120.2	941.6	−979
GE(W/O)	−90.4	5.7	122.4	982.3^a^	−974.5
GE	−91.85	3.9	120.6	974.6^a^	−972.3
*16 Channel Scanners*					
GE(W/O)	−93.49	0.74	120.33	952.01	−980.2
GE	−94.25	0.32	119.92	950.38	−979.63
Philips(W/O)	−88.3	5.9	130.2	855	−994.6
Philips	−87.6	6	130.4	854.3	−994.2
Siemens(W/O)	−90.4	2.1	127.1	920.5	‐1021^a^
Siemens	−99.4	1.5	126.7	919.8	‐1020.6^a^
Toshiba(W/O)	−97.6	−0.1	118.4	912.9	−997.9
Toshiba	−98.1	−0.2	118.3	912.6	−997.9

^a^ Houndsfield unit measures are beyond the ACR acceptable ranges for materials in Gammex 464 phantom.

**Table 4:** Helical Abdomen protocol HU values with and without (W/O) Extension Plates.

	*Polyethylene*	*Solid Water*	*Acrylic*	*Bone*	*Air*
*64 Channel Scanners*					
GE(W/O)	−88.16	9.05^a^	125.05	936.23	−976.96
GE	−91.58	4.35	121.66	929.03	−976.49
Philips(W/O)	−87.6	5.1	127	905	−991.9
Philips	−89.3	2.2	125.4	894.2	−987.8
Siemens(W/O)	−88.5	2	124.7	872.1	−992.9
Siemens	−88.8	0.7	124.7	873.2	−992.5
Toshiba(W/O)	−97	6	123	987.8^a^	‐1015^a^
Toshiba	−97.3	4.5	121.8	989.1^a^	‐1016.5^a^
GE(W/O)	−87.1	8.5^a^	127.5	950.6	−979.6
GE	−90.7	4.1	121	923.8	−973.6
*16 Channel Scanners*					
GE(W/O)	−90.49	3.56	123.82	889.15	−976.06
GE	−90.72	3.37	123.59	887.5	−974.34
Philips(W/O)	−90.6	1.9	123.3	892.7	−985.6
Philips	−91.42	0.59	121.15	890.21	−983.46
Siemens(W/O)	−81.2 ^a^	3.2	127.4	876.6	−985.3
Siemens	−82.9 ^a^	0.6	131	875.6	−994.4
Toshiba(W/O)	−99.8	0.6	116.8	963.7	−990.2
Toshiba	−101.3	−1.9	115	961.3	−988.1

^a^ Houndsfield unit measures are beyond the ACR acceptable ranges for materials in Gammex 464 phantom.

**Table 5:** ACR acceptable ranges for materials in Gammex 464 phantom.

	*Upper Limit (HU)*	*Lower Limit (HU)*
Polyethylene	−84	−107
Water	7	−7
Acrylic	135	110
Bone	970	850
Air	−970	−1005

## III. RESULTS

All nine scanners passed the water HU evaluation using the vendor's supplied phantom and testing procedure. Based on the HU measurements of specific materials within the ACR phantom, calibration issues were identified on four scanners. These failures were identified both with and without the Extension Plates.

HU values for 16‐ and 64‐channel scanners, axial head protocol, are shown in Table 3. HU values for bone failed for the GE VCT while Philips, Siemens, and Toshiba 64‐channel scanners all passed the bone HU evaluation. The GE, Philips, and Toshiba 16‐channel scanners passed the air HU evaluation, and the Siemens scanner failed the air HU. The pass/fail results for the HU values for all axial head protocols were unchanged by the presence of the extension plates.

Results for the 16‐ and 64‐channel scanners using the helical abdomen protocols from Table 2, are shown in Table 4. Bone and air HU values failed for the Toshiba 64‐channel scanner. GE, Philips, and Siemens 64‐channel scanners all passed the bone and air HU evaluation. Failure of the solid water HU value was noted in each of the GE VCT 64‐channel scanners without the Extension Plates in place. Using the Extension Plates, the HU values were within the allowable range for solid water.

Polyethylene HU values failed for the Siemens 16‐channel scanner. GE, Philips, and Toshiba 16‐channel scanners all passed the polyethylene HU evaluation. The Extension Plates had no positive or negative effect on the HU values utilizing the helical abdomen protocol with 64‐channel scanners in the Philips, Siemens, or Toshiba scanners. In addition, the Extension Plates had no positive or negative effect on the HU values for any of the 16‐channel scanners tested.

## IV. DISCUSSION

Calibration issues affecting the HU measure in CT may be due to multiple issues. We hypothesize that the probable cause of the calibration issue is the beam hardening correction and beam filtration, as the failure is replicated on two GE VCT 64‐channel scanners from independent clinical sites, showing a failure of the HU for bone in both scanners under the axial head protocol. Earlier work by Cropp supports this hypothesis.^(5)^ It should be noted that the Extension Plates did not mask the calibration‐related failures; HU values were inaccurate both with and without the Extension Plates in place.

Improvement in the solid‐water HU values were realized with the use of Extension Plates on all 64‐channel scanners evaluated using the helical abdominal protocol. The GE VCT solid‐water HU values improved enough to meet the ACR accreditation criteria. The data collected do not suggest a substantial benefit with other vendors' 64‐channel scanners or any 16‐channel scanner included in the study. However, they show no negative effect on HU values when used on those platforms. The addition of Extension Plates does not appear to mask calibration issues or cause inaccuracies in data collected while in use. Our data support the use of the Extension Plates for the GE VCT 64‐channel scanners as an effective means of addressing the known solid‐water HU assessment issue in helical mode using a 40 mm beam width. Additional investigation into the utility of Extension Plates on other wide‐beam CT scanners is needed. In particular, other wide‐beam GE scanners that also experience this issue may benefit from the use of the Extension Plates.

The Gammex 464 phantom and the Gammex 464‐EXTPLT‐KIT Extension Plates allow for a more efficient and accurate positioning of the phantom when compared with positioning the Gammex 464 phantom and a water phantom. The phantom/extension plate convenience could also be improved through the addition of a carrying case for both components of the system and extended cradle.

## ACKNOWLEDGMENTS

Alexandro Dell'Aiera, Ann Kirkwood, Ashley Litterbrand, Christina Quagliotti.

## Supporting information

Supplementary Material
